# Zinc Metal Complex of *tert*-butyl Substituted Phthalocyanine: Assessment of Photosensitizer Potential with Theoretical Calculations

**DOI:** 10.1007/s10895-025-04281-3

**Published:** 2025-03-29

**Authors:** Emre Güzel

**Affiliations:** Department of Engineering Fundamental Sciences, Faculty of Technology, Sakarya University of Applied Sciences, Sakarya, 54050 Turkey

**Keywords:** Phthalocyanine, Photophysic, DFT, Spin-orbit coupling, Singlet state, Triplet state

## Abstract

Photodynamic therapy (PDT) is a medical treatment that uses a photosensitizer molecule and a light source to induce the formation of reactive oxygen species in tissue. Phthalocyanines (Pcs) are macrocyclic photosensitizers with useful photophysical and photochemical properties in PDT applications. Motivated by these facts, this study has experimentally and theoretically investigated the suitability of 2,6-di-(tert-butyl)-4-methylphenoxy substituted zinc Pc (dt-ZnPc) in the first steps of PDT applications. Photophysical and photochemical characteristics of dt-ZnPc were measured and validated with the literature. Also, the suitability of the dt-ZnPc complex substituted with 2,6-di-(tert-butyl)-4-methylphenoxy groups for PDT applications has been supported by theoretical calculations for the first time. Density Functional Theory (DFT) and time-dependent DFT (TDDFT) calculations, incorporating both vertical excitation and adiabatic processes, were employed to elucidate the optical and photochemical mechanisms underlying singlet oxygen generation by unsubstituted metal-free and zinc Pcs and dt-ZnPc. Calculated excited state energies agreed well with experimental results, enabling the prediction of photosensitizer activity based on excited state properties and enhanced spin-orbit coupling (SOC) imparted by the metal center.

**Supplementary Information:**

The online version contains supplementary material available at 10.1007/s10895-025-04281-3.

## Introduction

Phthalocyanines (Pcs), an important class of macrocyclic compounds, have many uses in various application areas such as gas sensor [1], catalyst [2], hole transport material [3], and photosensitizer (Ps) [4–8]. Photodynamic therapy (PDT) is a minimally invasive treatment method used to treat cancer with light, without the need for surgery, and has attracted great interest in recent years. PDT uses Ps, after stimulation with light of the appropriate wavelength, to react with molecular oxygen to generate reactive oxygen species in the target tissue, causing cell death [4, 6]. Compared to conventional treatment modalities, PDT offers greater selectivity against tumor cells by preferentially using Ps that can be localized in tumor lesions and precisely delivering light of the appropriate wavelength to these lesions [9]. PDT therapy involves the application of a Ps (topical or intravenous) that selectively accumulates in tumor tissue, followed by exposure to light of the appropriate wavelength (usually in the red spectral region, λ ≥ 600 nm). Ps has the ability to transfer energy from light to molecular oxygen to form reactive oxygen species (ROS) such as singlet oxygen (^1^O_2_), superoxide radical (O_2_^-•^), hydroxyl radical (HO^-•^), and hydrogen peroxide (H_2_O_2_). These cytotoxic products initiate many biochemical processes that lead to damage and death of the target cancerous tissue. The activation of molecular oxygen via energy transfer requires the filling of the triplet-state manifold of the drug. The existence of effective intersystem crossing (ISC) pathways makes this possible. Nonetheless, ^1^O_2_ generation competes with alternative photophysical or photochemical pathways, including photoreactivity and luminescence through fluorescence or phosphorescence, which may inhibit the population of the triplet-state manifold. ISC, as a formally spin-forbidden activity, often renders the population of long-lived triplet states the rate-limiting phase for photophysical processes that result in the production of ^1^O_2_ [10]. The efficacy of ISC will significantly rely on the energy difference between triplet and singlet potential energy surfaces, as well as the presence of substantial spin − orbit coupling (SOC) matrix elements [11]. Moreover, the existence of extensive areas of singlet − triplet quasi-degeneracy on the potential energy surface landscape, where the system may become confined, is advantageous for enhancing ISC probability, even with minimal SOC, as observed in benzophenone [12].

Pcs are one of the most important macrocyclic Ps suitable for PDT applications. Pcs absorb light strongly in the 600–800 nm region, allowing light penetration into human tissues for effective PDT. In particular, zinc and aluminum metal coordinated Pc complexes have been used in the PDT of cancer due to closed shell; diamagnetic ions (Zn^2+^, Al^+ 3^) exhibit both high triplet quantum yields and long lifetimes. The chemical structure of the substituent groups attached to phthalocyanine’s skeletal structure significantly changes Pc’s properties, such as its solubility and electronic absorption properties [13, 14]. Conforming to their substituent positions, two tetra-substituted phthalocyanines may exhibit significant differences in their photophysical, and photochemical behavior can be distinguished. However, the solubility of phthalocyanines is very poor and most of their application needs to be investigated in soluble form. Pcs can generally be solubilized only in concentrated polar solvents such as concentrated sulfuric acid or DMF, DMSO, pyridine and THF; therefore, the use of phthalocyanines in a variety of application areas is becoming very limited [7, 15]. In this manner, the synthesis of new phthalocyanine systems should be designed so that the final phthalocyanine derivatives will become soluble enough to perform the desired applications.

Taking these properties into consideration, it has focused on highly soluble photosensitizers as a strategy to explain Pcs in conjunction with the 2,6-di-(tert-butyl)-4-methylphenoxy group to study photophysicochemical properties and detailed theoretical calculations. In the literature, although there are many studies on phthalocyanines with tert-butyl groups [16–20], the number of detailed theoretical studies investigating the photosensitizer potential of phthalocyanines containing these groups is very few [21, 22]. In addition to experimental measurements, a comprehensive theoretical analysis was carried out using density functional theory (DFT). Using time-dependent DFT (TDDFT) calculations, the photophysics of Pcs were investigated through both vertical excitation and adiabatic processes. Furthermore, the spin-orbit coupling (SOC) effect was included in the calculations and a study of the electronic properties of Pcs for efficient ROS production was carried out.

**Fig. 1:**
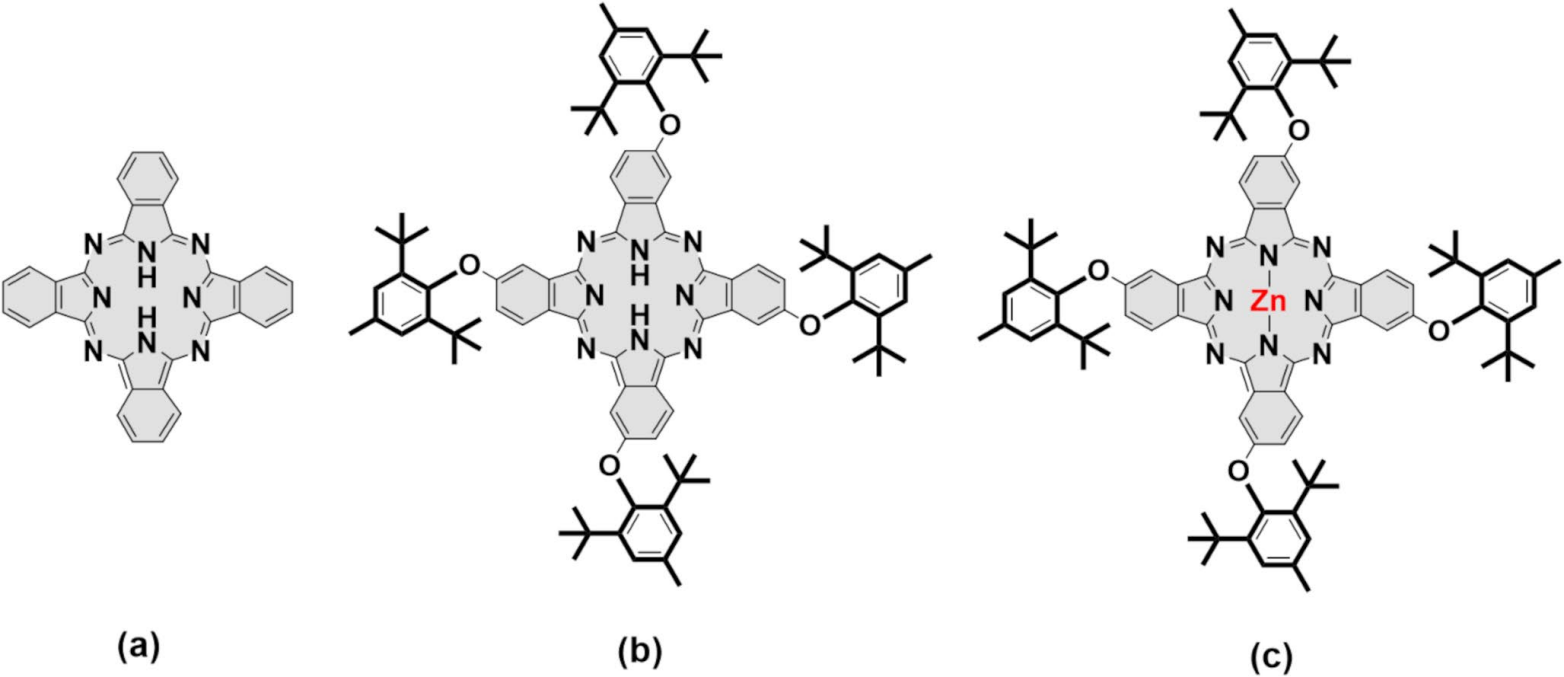
Molecular structure of unsubstituted metal-free (H_2_Pc) (**a**), tetra 2,6-di-(tert-butyl)-4-methylphenoxy substituted metal-free phthalocyanine (dt-H_2_Pc) (**b**), tetra 2,6-di-(tert-butyl)-4-methylphenoxy substituted zinc phthalocyanine (dt-ZnPc) (**c**)

## Experimental

Figure 1 shows the molecular structure of 2,6-di-(tert-butyl)-4-methylphenoxy substituted zinc Pc. Starting compound 4-(2,6-di-tert-butyl-4-methylphenoxy)phthalonitrile and its zinc phthalocyanine (dt-ZnPc) were prepared in two facile steps according to previous works [23, 24]. The photophysical, photochemical, and theoretical parameters were presented in supporting information.

## Results and Discussion

### Photophysical and Photochemical Studies

**Fig. 2:**
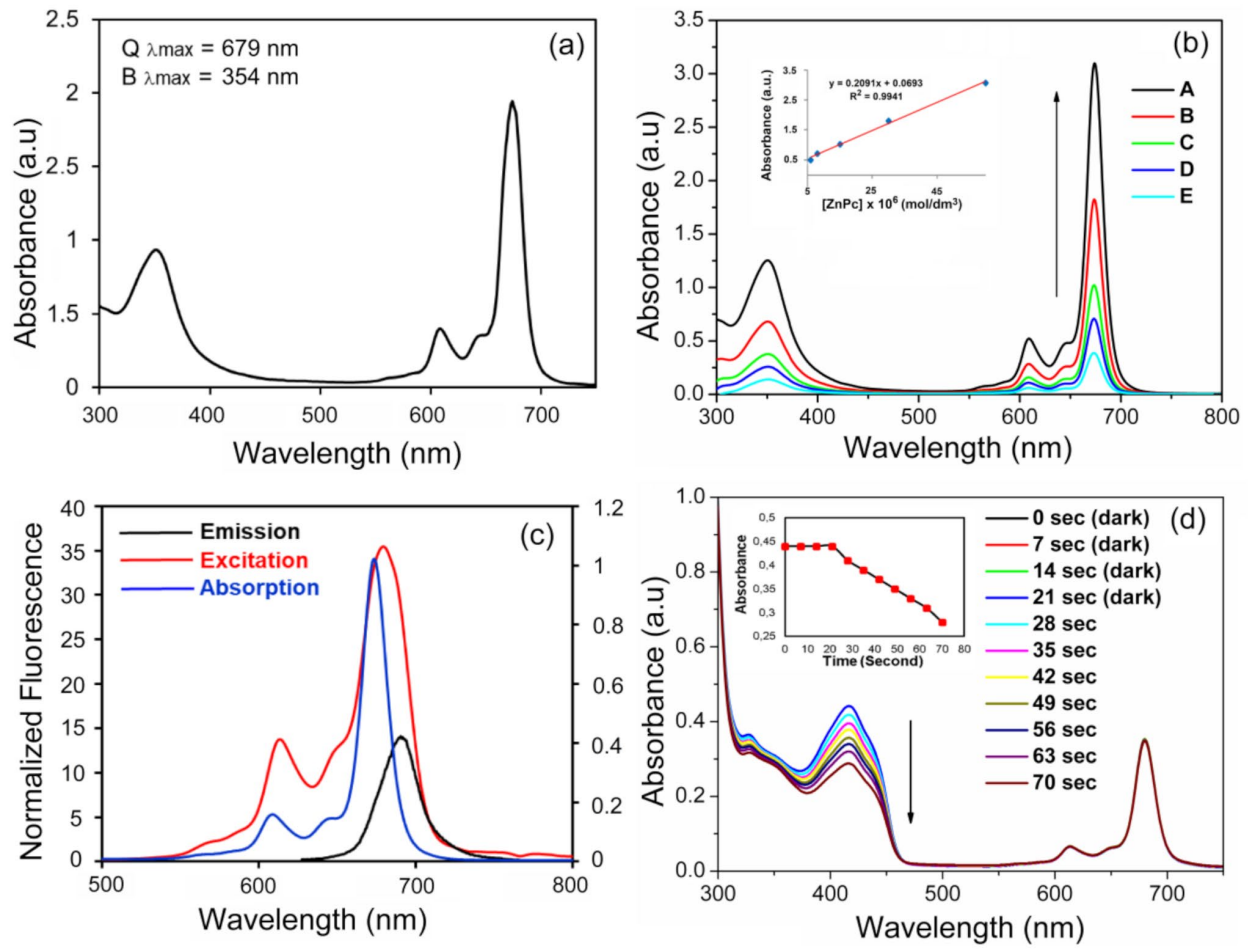
(**a**) Absorption spectrum of dt-ZnPc in DMSO (9 × 10^− 6^ mol dm^− 3^). (**b**) Electronic absorption spectra of dt-ZnPc in DMSO at different concentrations: (A) 16 × 10^− 6^, (B) 8 × 10^− 6^, (C) 4 × 10^− 6^, (D) 2 × 10^− 6^, (E) 1 × 10^− 6^ mol dm^− 3^. The inset shows a calibration plot for the Q band maximum. (**c**) Electronic absorption, fluorescence emission, and excitation spectra of dt-ZnPc in DMSO. (**d**) The reaction of the singlet oxygen generated by photosensitization with 1,3-diphenyl-isobenzofuran (DPBF) in DMSO in the presence of dt-ZnPc. First 21 s, the solution was left in the dark.; after that, it was irradiated with a light source (650 nm cut on filter) for 49 s. The inset shows the plot for DPBF absorbance versus irradiation time

UV-Vis spectra of the Pcs exhibit distinctive absorptions in the B band region (UV region) nearly 350–360 nm, resulting from the deeper π–π* transitions, and in the Q-band region nearly 680–700 nm, which is related to the π–π* transition from the HOMO to the LUMO of the Pc ring [25]. The UV-Vis absorption spectrum of deeply green-colored peripherally substituted dt-ZnPc in DMSO shows two main peculiarities as expected, the characteristic ligand centered π-π* transitions of a zinc Pc with the Soret or B-band and Q-band maxima at 354 nm (log ε = 4.92) and 679 nm (log ε = 5.13), respectively (Fig. 2a).

The superposition of monomers, dimers, and rings in the solvent medium is a common way to describe aggregation [4, 26, 27]. Phthalocyanine aggregation is influenced by a wide range of factors, including concentration, solvent type, substituents at the periphery and/or non-periphery, metal ions at the macrocyclic core, and operating temperature. In order to better examine the solubility and aggregation properties of the complexes, the dilution study was also carried out at ambient temperature in DMSO. The absorption spectra of dt-ZnPc were presented at different concentrations (Fig. 2b). Because of the formation of aggregate species, a new remarkable band did not emerge on the high energy side as concentration increased, and the Q band’s absorption intensity was in agreement with Lambert-Beer’s law (inset of Fig. 2b).

Assessing fluorescence behavior and fluorescent quantum yields are the first steps toward identifying a photosensitizer for photodynamic treatment. In this context, the fluorescence emission, excitation, and absorption spectra of ZnPc were examined in DMSO (Fig. 2c). The studied dt-ZnPc showed similar electronic absorption and fluorescence excitation spectra. The Stokes shift (λ_ems_–λ_ex_) value of dt-ZnPc was observed at 9 nm, the same region as the standard phthalocyanines. The fluorescence emission maximum was monitored at 688 nm for dt-ZnPc in DMSO. The fluorescence quantum yield of dt-ZnPc was determined using a comparative method. The standard reference unsubstituted ZnPc (Φ_F_ = 0.18 [28]) was used to study dt-ZnPc in DMSO. The Φ_F_ value of the studied ZnPc was found 0.19 in DMSO solution and this result was validated in the literature [23]. The 2,6-di-(tert-butyl)-4-methylphenoxy group containing dt-ZnPc showed nearly the same Φ_F_ value with reference unsubstituted ZnPc.

Diphenylisobenzofuran (DPBF) is recognized for its ability to quench singlet oxygen, forming endoperoxide species in solution medium and by chemical addition reactions. The singlet oxygen capacity of dt-ZnPc was investigated in DMSO using a similar previous method [27]. Detailed control studies have been conducted to eliminate additional potential media sources that may influence the decline in absorption. Spectral changes during singlet oxygen determination for dt-ZnPc are presented in Fig. 2d. No significant change was observed in the absorption spectrum of dt-ZnPc when kept in the dark for the first 21 s. Moreover, the absorption peak of the DPBF, ^1^O_2_ quencher compound, disappeared rapidly within 56 s in red light irradiation (with 650 nm cut-off filter) on dt-ZnPc solution. The Φ_Δ_ value of dt-ZnPc was found 0.57 in the DMSO solution. The dt-ZnPc containing the 2,6-di-(tert-butyl)-4-methylphenoxy groups showed a slightly lower Φ_Δ_ value with reference unsubstituted ZnPc in DMSO.

### Computational Process

Density functional theory (DFT) [29, 30] calculations were performed using the B3LYP exchange-correlation functional and the 6-31G(d), 6-31G(d, p), and 6-311 + + G(2d,2p)//Zn/*gen*/6-31G(d, p) basis sets (6-311 + + G(2d,2p) was applied to the dt-H_2_Pc side, while the Zn atom was treated by the *genecp* approach with the 6-31G(d, p) basis set) using Gaussian 09 software [31]. The calculations were carried out without imposing any symmetrical constraints on the molecular structures. The resulting geometries were determined to correspond to the global minima on the potential energy surface, as confirmed by the absence of imaginary frequencies in the vibrational frequency calculations. The electronic parameters of the compounds were derived from gas-phase, dimethyl sulfoxide (DMSO), and Tetrahydrofuran (THF) phase calculations conducted at B3LYP/6-31G(d) and B3LYP/6-31G(d, p) level. Global chemical reactivity parameters, including chemical hardness, electronegativity, and the electrophilic index, were computed using frontier molecular orbital (FMO) energy eigenvalues.

In accordance with the experimental processes, TDDFT calculations were also carried out in DMSO and THF phases employing the 6-31G(d) and 6-31G(d, p) basis sets, and the conductor-like polarizable continuum model (CPCM) was used for solution-solvent interaction in these processes. Moreover, calculations were also performed using the higher-level 6-311 + + G(2d,2p)//Zn/*gen*/6-31G(d, p) basis set in the THF phase. In order to determine the spectroscopic properties of electronic transitions, vertical and adiabatic excitation energies were separately calculated and analyzed. Vertical excitation energies were determined using single-point calculations at the ground-state geometry within the framework of the Franck-Condon approximation. On the other hand, adiabatic excitation energies were computed at the B3LYP/6-31G(d) level of theory by optimizing the geometry of the S_1_ state (in both THF and DMSO environments), accounting for nuclear relaxation in the excited state [32, 33]. Also, spin-orbit coupling (SOC)-TDDFT calculations were carried out in the THF phase with the B3LYP/6-31G(d) level of theory using ORCA software [34, 35].

#### Theoretical Analysis

**Fig. 3:**
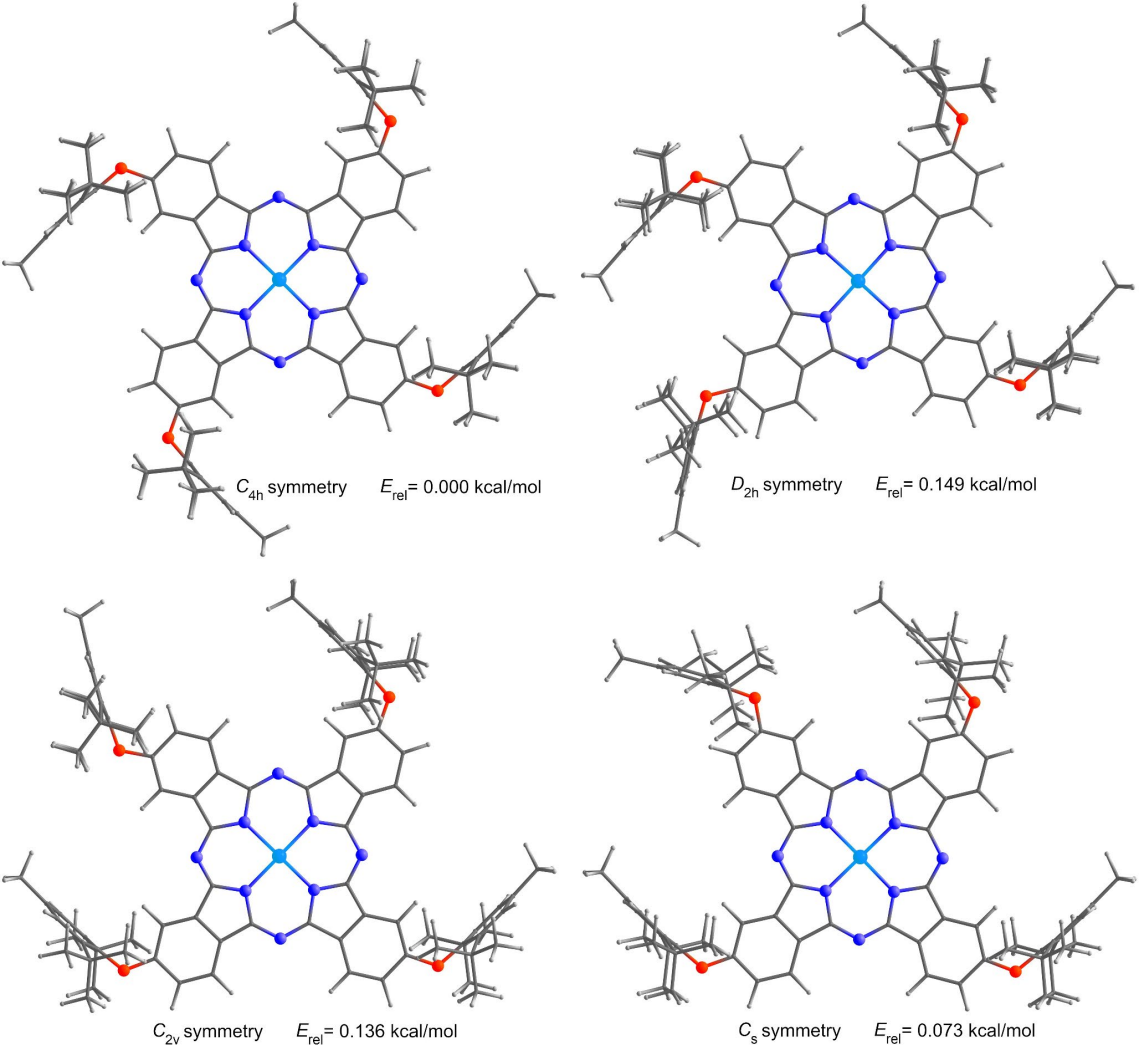
Optimized molecular structures of possible isomeric forms of dt-ZnPc. Relative electronic energies were calculated by B3LYP/6-31G(d) level of theory in gas phase

The optimized molecular structures and relative energies of dt-ZnPc in its four possible isomeric forms (C_s_, C_2v_, C_4h_, and D_2h_, based on the position of the substitutions) are shown in Fig. 3. In addition to the symmetric nature of the 2,6-di-(tert-butyl)-4-methylphenoxy fragments at the 2 and 6 positions of the benzene rings, the interaction of the central nitrogen atoms of the Pc with Zn, along with the contribution of the aza-bridge, resulted in the maintenance of a planar geometry on the Pc skeleton (see supplementary data S1-S2). It can be said that the preservation of the planar geometry of dt-ZnPc in the symmetry groups causes the relative electronic energies between these symmetries to have values ​​close to each other.

**Fig. 4:**
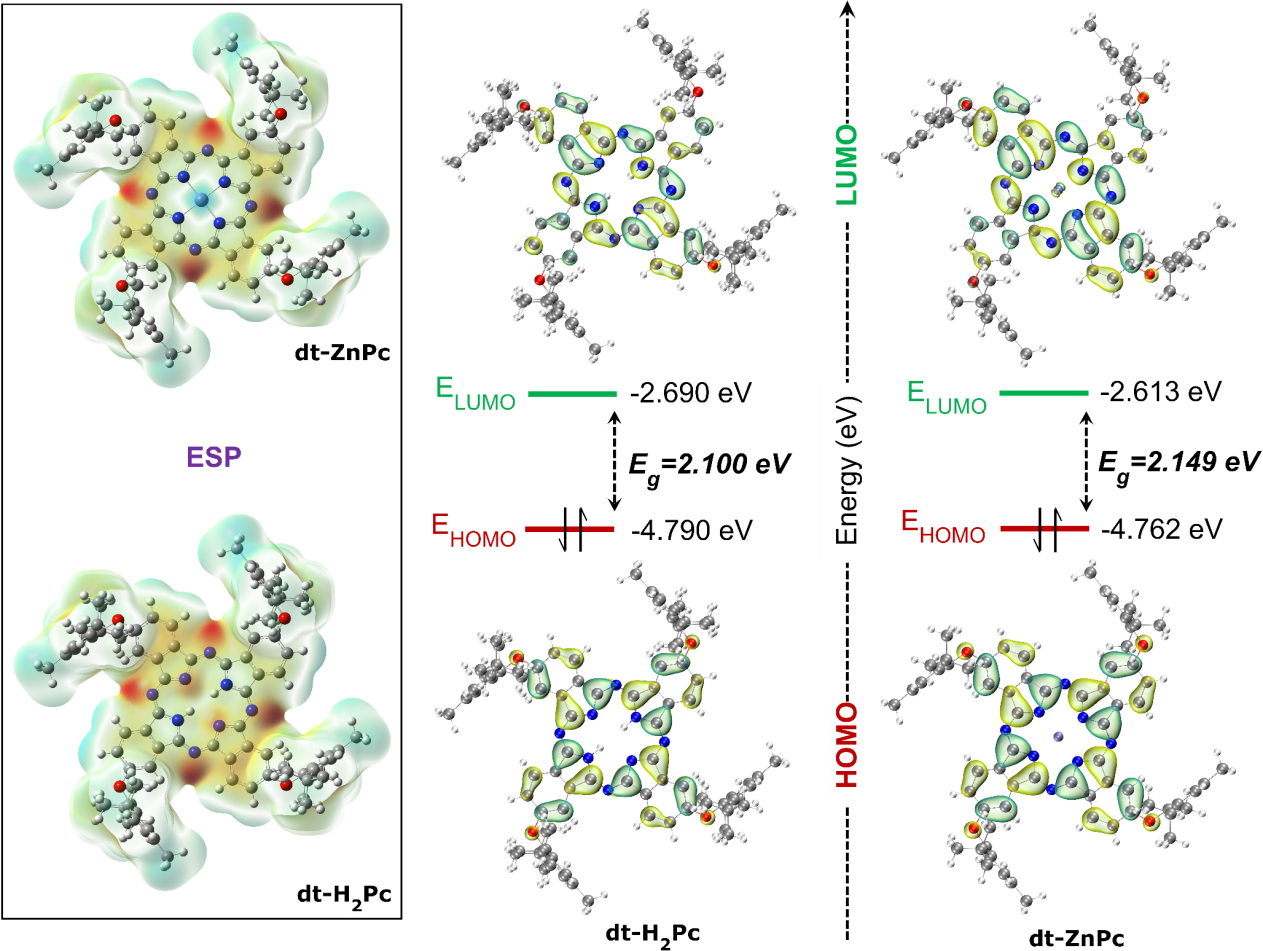
Electrostatic potential (ESP) maps and Molecular orbitals (HOMO-LUMO) of dt-H_2_Pc and dt-ZnPc, calculated by 6-31G(d, p) basis set in gas phase

The frontier molecular orbitals (FMO), HOMO (Highest Occupied Molecular Orbital), and LUMO (Lowest Unoccupied Molecular Orbital), which are fundamental quantum parameters influencing optical and electrical properties, were calculated using the same methods, and subsequently, the HOMO-LUMOs were plotted. Their corresponding energy values were determined (Fig. 4; Table 1). In both dt-ZnPc and metal-free Pc (dt-H_2_Pc), the HOMO exhibits similar localization patterns on the phthalocyanine macrocycle, while the LUMO is localized on the non-planar Pc with etheric structure except for the two phenyl rings of the Pc.

Table 1 summarizes the electronic parameters of the compounds obtained using different computational levels in the gas phase, DMSO, and THF solvents. Gas-phase calculations indicate that dt-H_2_Pc has a lower energy band gap compared to H_2_Pc (2.148 eV for H_2_Pc vs. 2.100 eV for dt-H_2_Pc). This indicates the effect of the tetra 2,6-di-(tert-butyl)-4-methylphenoxy substituent on the energy band gap (E_g_) values of the compounds. For dt-ZnPc, the BS1 and BS2 methods in the gas phase yield similar results, while the higher-level BS3 method resulted in a lower band gap (2.126 eV). A similar trend is observed in calculations performed in DMSO and THF solvents. In addition, the binding of Zn to dt-H_2_Pc provided a larger difference in the LUMO energy of the compound compared to the HOMO energy, and Zn strengthened the nucleophilic property of the compound. This result is also seen from the calculated electrophilic index (ω) and nucleophilic index (𝛆) values ​​of the phthalocyanines. It was calculated that the electronegativity of H_2_Pc decreased with the bonding of substituted groups and reached a minimum value in the dt-ZnPc compound (3.681 eV for BS1), supporting the notion that dt-ZnPc exhibits enhanced nucleophilic character due to its increased propensity for electron donation. Calculations on dt-ZnPc in DMSO and THF phases to investigate solvent effects revealed that, while the E_g_ remained largely unaffected by the solvent (0.001–0.003 eV change), both the HOMO and LUMO energy levels decreased (from 0.175 to 0.147 eV for E_HOMO_, and from 0.176 to 0.149 eV for E_LUMO_). In addition, it was observed that solvents increased the electronegativity and electrophilic index of dt-ZnPc while decreasing the nucleophilic index (Table 1).

**Table 1:** Calculated electronic parameters of compounds in gas, DMSO, and THF phases

Solv.	Comp.	Meth.	$\:{\varvec{E}}_{\mathbf{H}\mathbf{O}\mathbf{M}\mathbf{O}}$ (eV)	$\:{\varvec{E}}_{\mathbf{L}\mathbf{U}\mathbf{M}\mathbf{O}}$ (eV)	$\:{\varvec{E}}_{\varvec{g}}$ (eV)	$\:\varvec{\eta\:}$ (eV)	$\:\chi\:$ (eV)	ω (eV)	𝛆 (eV)	ω^+^ (eV)	ω^−^ (eV)
Gas phase	H_2_Pc	BS1	-4.977	-2.829	2.148	1.074	3.903	7.092	4.518	1.863	5.766
		BS2	-4.985	-2.838	2.147	1.074	3.912	7.126	4.510	1.876	5.787
	dt-H_2_Pc	BS1	-4.783	-2.682	2.101	1.051	3.733	6.631	4.712	1.712	5.444
		BS2	-4.790	-2.690	2.100	1.050	3.740	6.661	4.705	1.723	5.463
	dt-ZnPc	BS1	-4.755	-2.606	2.149	1.075	3.681	6.303	4.740	1.580	5.261
		BS2	-4.762	-2.613	2.149	1.075	3.688	6.328	4.732	1.589	5.276
		BS3	-5.000	-2.874	2.126	1.063	3.937	7.292	4.495	1.943	5.881
DMSO	dt-ZnPc	BS1	-4.930	-2.782	2.148	1.074	3.856	6.922	4.565	1.802	5.658
		BS2	-4.934	-2.786	2.148	1.074	3.860	6.936	4.561	1.807	5.667
THF	dt-ZnPc	BS1	-4.906	-2.760	2.146	1.073	3.833	6.846	4.589	1.775	5.608
		BS2	-4.909	-2.762	2.147	1.074	3.836	6.852	4.586	1.777	5.612
		BS3	-5.127	-3.003	2.124	1.062	4.065	7.778	4.368	2.122	6.187

Solv.: Solvent, Meth.: Method, BS1: B3LYP/6-31G(d), BS2: B3LYP/6-31G(d, p), BS3: B3LYP/6-311 + + G(2d,2p)//Zn/*gen*/6-31G(d, p), E: Energy, $\:{\varvec{E}}_{\varvec{g}}$: $\:{E}_{LUMO}-{E}_{HOMO}$, $\:\varvec{\eta\:}$: Chemical Hardness, $\:\varvec{\chi\:}:\:$ Electronegativity, ω: Electrophilic index, 𝛆: Nucleophilic index, ω^+^: Electroaccepting power, ω^−^: Electrodonating power

**Fig. 5:**
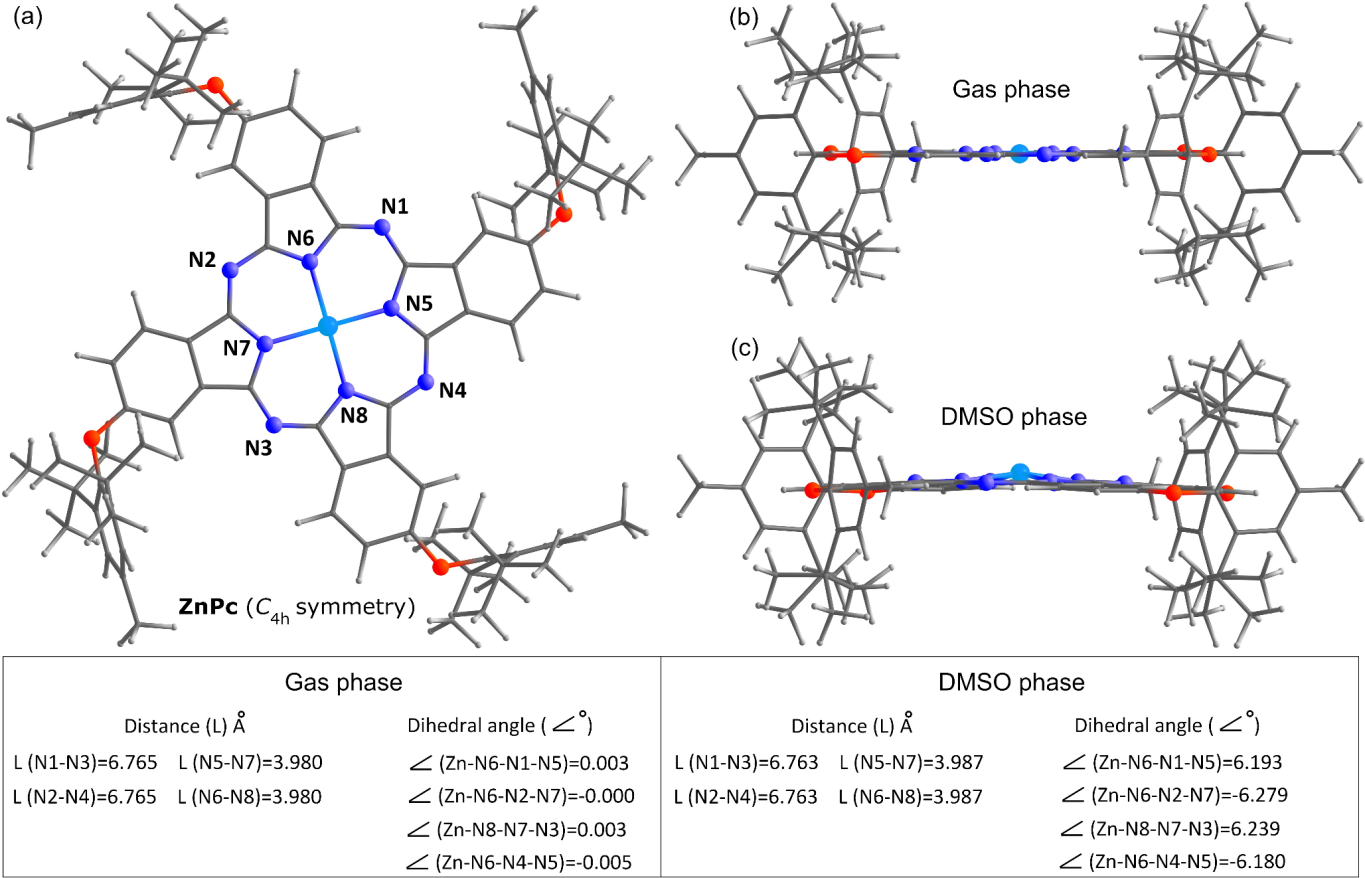
Effect of the solvent on the dt-ZnPc, calculated using 6-31G(d, p) basis set in gas and DMSO phase

The effect of the solvent on dt-ZnPc is shown in Fig. 5. In general, for both H_2_Pc and dt-H_2_Pc with substituted groups, the Pc skeleton remains approximately planar. As previously mentioned, this is primarily due to the symmetrical positioning of the di-(tert-butyl) fragment at the 2 and 6 positions of the benzene ring, which forces the benzene ring to be positioned perpendicular to the Pc plane. As seen in Fig. 5, the dihedral angles formed by the central nitrogen atoms and the aza-bridge nitrogen atoms with Zn are very close to zero (between 0.000° and 0.005°). In the isomeric forms of dt-ZnPc, these perpendicular planar conformations have made the isomeric structures low in energy. However, optimizations conducted in the solvent medium of dt-ZnPc show that the solvent causes Zn to be and displaced approximately 6.2° out of the plane dihedral. Furthermore, calculations in the THF phase revealed that the Zn atom is located approximately 5.8° to 5.9° out of the Pc plane dihedrally. The electronic parameters calculated for the dt-ZnPc conformation in the solvent are presented in Table 1, and since the effect of the solvent (whether DMSO or THF) on HOMO and LUMO energies of dt-ZnPc is consistent and proportional, the HOMO-LUMO energy gap of dt-ZnPc remains almost unchanged, with differences of 0.001 eV and 0.002 eV for DMSO and THF, respectively, when using the 6-31G(d) and 6-31G(d, p) basis sets. However, a significant change was observed in the calculations using the higher-level 6-311 + + G(2d,2p)//Zn/*gen*/6-31G(d, p) basis set (see Table 1, TFH/BS3 data). Furthermore, DMSO, with its higher polarity and dielectric constant compared to THF, exhibited a more significant impact on χ, ω, and 𝛆 values of the dt-ZnPc.

**Fig. 6:**
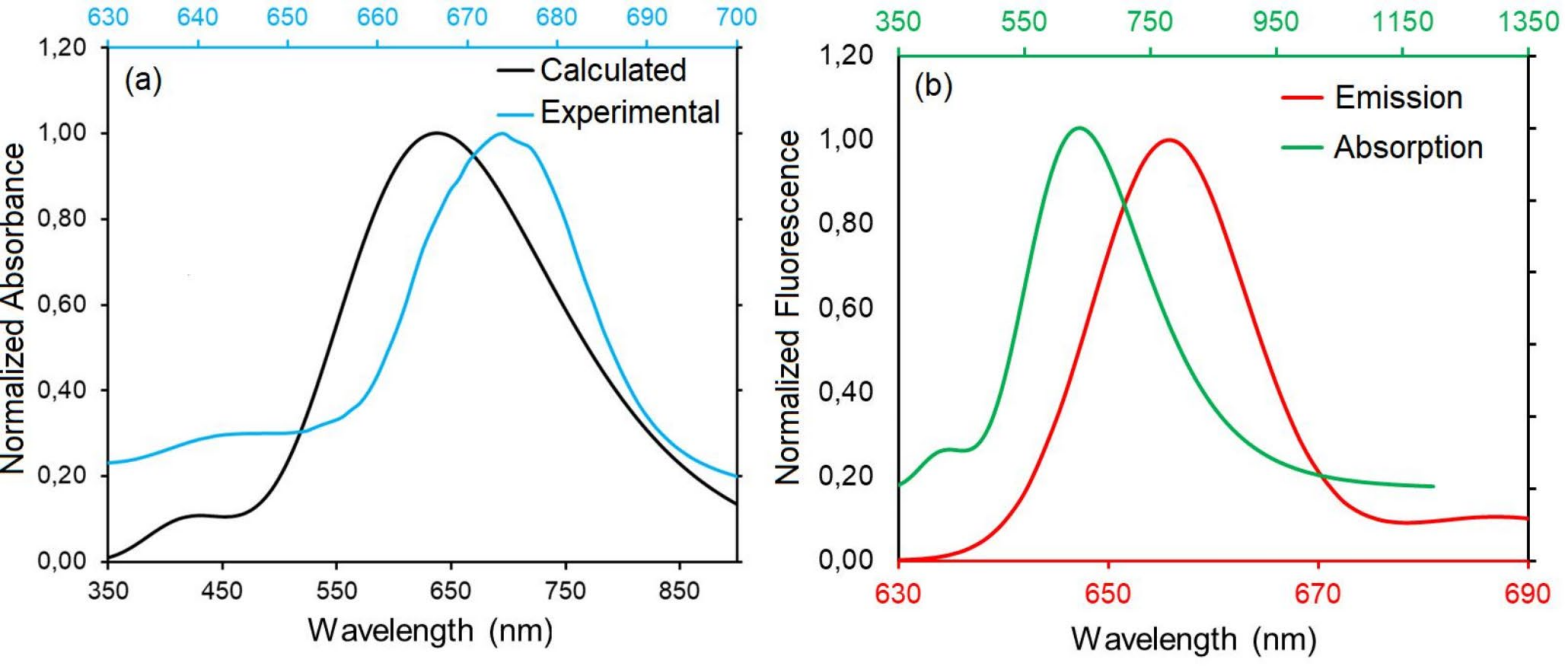
(**a**) Calculated and experimental absorption spectra of dt-ZnPc in DMSO phase (**b**) Calculated absorption and emission spectra of dt-ZnPc in gas-phase

To calculate the transition energies to the excited electronic states of dt-ZnPc, vertical excitation energies based on the Franck-Condon approach were performed using the basis sets 6-31G(d), 6-31G(d, p), and 6-311 + + G(2d,2p)//Zn/*gen*/6-31G(d, p). Comparison of the theoretical results with experimental UV-Vis spectroscopy data showed a good agreement between the calculated and observed transition energies. The TDDFT-calculated absorption spectra showed minimal variation between the DMSO and THF solvent environments, indicating that solvent choice did not significantly affect the UV-Vis calculations. TDDFT calculations of the S0→S1 transition in DMSO and THF, employing the 6-31G(d) and 6-31G(d, p) basis sets, yielded Q-band absorption peaks around 1.944–1.945 eV (637.35–637.78 nm). These calculated values are in reasonable agreement with the experimental Q-band peak at 1.825 eV (679 nm) (Fig. 6a), with a difference of 0.12 eV. The combined use of the larger 6-311 + + G(2d,2p) basis set for non-Zn atoms and the 6-31G(d, p) basis set for Zn yielded calculations that better align with experimental data, predicting the Q-band peak wavelength at 648.3 nm with an energy deviation of only 0.088 eV. In contrast, gas-phase calculations, conducted without accounting for solvent effects, produced results more rapidly but with a larger deviation of 0.19 eV from the experimental values (see Supplementary Figure S1 for comparative UV data).

TDDFT calculations (in gas-phase), performed using the adiabatic approximation, predicted absorption at 640.3 nm and fluorescence emission at 655.4 nm in the Q-band region. This prediction is consistent with the expected Stokes shift and shows overall agreement with experimental data. The experimentally observed Q-band maximum is located at 679 nm, exhibiting a bathochromic shift of 24 nm relative to the theoretical prediction (Fig. 6b). The minor discrepancy can be attributed to limitations inherent in the functionals and basis sets employed in the calculations, as well as constraints of the adiabatic approximation, and the challenges associated with explicitly including solvent-solute interactions and clustering phenomena in the calculations.

Although there are many transitions between HOMO and LUMO, calculations indicate that the predominant transitions in the Q band region are HOMO → LUMO and HOMO-1 → LUMO. The contributions of these transitions, evaluated using the 6-31G(d) and 6-31G(d, p) basis sets in both DMSO and THF phases, were approximately 97% for HOMO → LUMO and between 64 and 85% for HOMO-1 → LUMO. It was observed that the contributions of substituents in these transitions were more pronounced compared to those in the HOMO → LUMO transitions.

The potential of the dt-ZnPc for PDT relies on efficient energy transfer between the photosensitizer’s excited triplet state and molecular oxygen. Understanding this process requires exploring the photophysical pathways leading from excited singlet states to populate the triplet state. The thermodynamic favorability of these pathways necessitates a lower energy for the triplet state compared to the excited singlet state, with a minimal energy gap between them. Subsequently, based on the lower-energy singlet and triplet states, the most probable intersystem crossing (ISC) pathways were investigated.

In this work, using the TDDFT approach, it was calculated both vertical and adiabatic excitation energies for the singlet (S1) and triplet (T1) states of dt-ZnPc, as these distinct approaches are crucial for comprehending molecular behavior post-excitation, particularly fluorescence (see Supplementary Table S1-S4 for calculated data). Vertical excitation energies calculated in DMSO phase, representing Franck-Condon transitions, were calculated as S1 = 1.944 eV and T1 = 1.116 eV (1110.93 nm) (see Table S1). In contrast, adiabatic excitation energies, accounting for ground and excited state geometry relaxation, yielded S1 = 1.663 eV (745.43 nm) and T1 = 0.839 eV (1477.91 nm) (see Table S3). As expected, adiabatic energies were consistently lower due to structural relaxation, stabilizing the excited state. The larger energy difference between vertical (0.828 eV) and adiabatic (0.824 eV) excitations for S1-T1 indicates a greater degree of geometrical relaxation in the singlet state upon excitation. This observation aligns with the generally slower dynamics and longer lifetimes associated with triplet states, suggesting less geometrical distortion during T1 excitation. Simulating the fluorescence emission spectrum in the gas phase, our calculations accurately predicted a fluorescence emission energy of 1.892 eV (655.4 nm), closely matching the experimental value of 1.802 eV (688 nm) with a deviation of only 0.09 eV. Also, the localization of a metal atom into the central cavity of the dt-ZnPc structure has a pronounced effect on the energy gap between the S1 and T1 states. Specifically, our calculations reveal a significant reduction in the S1-T1 energy difference compared to the metal-free configuration. In the dt-H_2_Pc system, the S1-T1 gap is calculated to be 0.917 eV (see Table S1), highlighting the influence of the metal on the electronic structure and spin states of the molecule. The reduction in the S1-T1 energy difference can be attributed to enhanced spin-orbit coupling (SOC) introduced by the metal atom, facilitating the overlap between singlet and triplet states. As a result, the metal insertion promotes intersystem crossing (ISC) and allows for a more efficient transition between S1 and T1. The SOC calculations reveal that the incorporation of a zinc atom into the Pc system reduces the S1-T1 energy difference from 0.919 eV in the dt-H_2_Pc to 0.830 eV in the dt-ZnPc compound (see Table S4). Additionally, these calculations demonstrate that Zn incorporation into the dt-H_2_Pc system reduces the S1-T1 energy gap, enhancing intersystem crossing and triplet state formation, which increases singlet oxygen generation. These results align with the experimental data, confirming dt-ZnPc’s role in boosting fluorescence quenching and singlet oxygen production.

## Conclusion

In a nutshell, it was demonstrated that the attachment of 2,6-di-(tert-butyl)-4-methylphenoxy substituent to the Pc core significantly enhanced the solubility, improving the functionality of zinc phthalocyanine (dt-ZnPc). Furthermore, the addition of a zinc atom to the Pc center effectively reduced the energy gap between the excited singlet state (S1) and the triplet state (T1); our spin-orbit coupling calculations strongly confirm this phenomenon. The calculations highlight the role of metal-induced spin-orbit interactions in modulating the excited state dynamics. In particular, the transition from the excited singlet state to the triplet state in dt-ZnPc was determined to be thermodynamically favorable and the presence of the metal atom increased the spin-orbit coupling and facilitated the transition, considering the vertical excitation and adiabatic processes. The calculated transition energies agree well with experimental UV-Vis and fluorescence spectroscopy data. Experimental and theoretical investigations show that dt-ZnPc exhibits remarkable photophysical properties for future studies, revealing their potential as photosensitizers for photodynamic therapy (PDT) applications.

## Electronic Supplementary Material

Below is the link to the electronic supplementary material.

Supplementary Material 1

## Data Availability

No datasets were generated or analysed during the current study.
