# Supplementary material for: Zinc Metal Complex of tert-butyl Substituted Phthalocyanine: Assessment of Photosensitizer Potential with Theoretical Calculations
Source: J Fluoresc. 2025 Mar 29;35(10):9791–800. doi: 10.1007/s10895-025-04281-3 (PMC12672670; doi:10.1007/s10895-025-04281-3)
Supplement: Supplementary file 1 — Supplementary Material 1 [file 10895_2025_4281_MOESM1_ESM.pdf]

Supporting Information

Zinc metal complex of *tert*-butyl substituted phthalocyanine: Assessment of photosensitizer potential with theoretical calculations

Emre Güzel *

Department of Engineering Fundamental Sciences, Faculty of Technology, Sakarya University of Applied Sciences, 54050 Sakarya, Türkiye

* Corresponding Author: Emre Güzel
E-mail address: eguzel@subu.edu.tr

1. **Photophysical and photochemical parameters**

At room temperature, fluorescence excitation and emission spectra were recorded on a Shimadzu spectrofluorometer using a 1 cm path-length cuvette. Fluorescence quantum yield (Φ_F) is determined in DMSO by the comparative method using equation **1**[1–3].

$$\Phi_F = \Phi_{F_{Std}} \frac{F*A_{Std}*\eta^2}{F_{Std}*A*\eta_{Std}^2} \quad \textbf{(1)}$$

where F and F_{Std} are the areas under the fluorescence emission curve of the sample and the standard, respectively. A and A_{Std} are the respective absorbances of the sample and standard at the excitation wavelengths, respectively. n^2 and n_{Std}^2 are the refractive indices of solvents used for the sample and standard, respectively. Reference (unsubstituted) ZnPc (Φ_F = 0.20) [4] was employed as the standard in DMSO. The solutions' absorbance at the excitation wavelength ranged between 0.04 and 0.05. Both the sample and standard were excited at the same wavelength. Singlet oxygen quantum yield (Φ_Δ) determination was carried out using the experimental set-up described in the literature[5, 6]. Typically, a 3 mL portion of the respective unsubstituted zinc (II) phthalocyanine (ZnPc) and the studied phthalocyanine solutions (C= $1 \cdot 10^{-5}$ M) containing the singlet oxygen quencher was irradiated in the Q band region with the photo-irradiation set-up described in references [7–9]. Singlet oxygen quantum yield (Φ_Δ) was determined in DMSO using the relative method with unsubstituted zinc (II) phthalocyanine (ZnPc) as a reference. DPBF was used as the chemical quencher for singlet oxygen in DMSO. Equation **2** was employed for the calculations:

$$\Phi_\Delta = \Phi_\Delta^{Std} \frac{R* I_{abs}^{Std}}{R^{Std} * I_{abs}} \quad \textbf{(2)}$$

where Φ_Δ^{std} is the singlet oxygen quantum yield for the standard unsubstituted zinc (II) phthalocyanine. R and R_{Std} are the DPBF photobleaching rates in the presence of studied phthalocyanine and standard, respectively. I_{abs} and I_{abs}^{Std} are the rates of light absorption by the studied phthalocyanine and standard, respectively. To avoid chain reactions induced by DPBF

in the presence of singlet oxygen, the concentration of quencher (DPBF) was lowered to ~5x10^{-6} M. Solutions of sensitizer (C= 1· 10^{-6} M) containing DPBF were prepared in the dark and irradiated in the Q band region using the photoirradiation setup. DPBF degradation at 417 nm was monitored. The light intensity 7.05 · 10^{15} photons s^{-1} cm^{-2} was used for determinations. The absorption band of DPBF is reduced by light irradiation.

2. DFT optimization data

Data S1. The calculated optimized geometries of dt-ZnPc.

**Optimized geometry of dt-ZnPc (in gas-phase// B3LYP/6-31G(d))**				**Optimized geometry of dt-ZnPc (in DMSO phase// B3LYP/6-31G(d))**			
C	4.596524	-4.741919	-0.004085	C	4.597836	-4.73984	-0.00144
C	3.484805	-5.614565	0.001372	C	3.486572	-5.61263	-0.00641
C	4.430644	-3.354856	-0.007927	C	4.431221	-3.35379	0.072873
C	2.186979	-5.119366	0.003087	C	2.189383	-5.1186	0.064148
C	3.123072	-2.871853	-0.005852	C	3.12499	-2.87196	0.141763
C	2.008289	-3.733736	-0.000536	C	2.010148	-3.73468	0.140161
C	2.591787	-1.509264	-0.00756	C	2.590632	-1.51001	0.206769
C	0.823509	-2.886879	0.000681	C	0.826496	-2.88535	0.207875
N	1.220545	-1.571024	-0.003415	N	1.223086	-1.57304	0.24748
N	-0.422057	-3.355365	0.00527	N	-0.42108	-3.35413	0.200928
N	3.356223	-0.421426	-0.011944	N	3.355681	-0.42023	0.192821
C	-1.509895	-2.59092	0.007579	C	-1.51083	-2.58902	0.214627
C	2.887733	0.824147	-0.012282	C	2.886941	0.827356	0.202776
N	-1.571658	-1.219672	0.006076	N	-1.57378	-1.22137	0.250923
C	-2.872477	-3.122193	0.013123	C	-2.87294	-3.12363	0.155882
C	-3.734357	-2.007398	0.015446	C	-3.73578	-2.00888	0.154106
C	-2.887507	-0.822623	0.010561	C	-2.88627	-0.82496	0.21491
C	-3.35548	-4.429764	0.015991	C	-3.35486	-4.43015	0.09365
C	-5.119979	-2.18607	0.021597	C	-5.12003	-2.18863	0.085253
C	-4.742539	-4.595627	0.021809	C	-4.7412	-4.59727	0.026725
C	-5.615178	-3.483894	0.024972	C	-5.61419	-3.48616	0.021954
N	-3.355996	0.42295	0.010302	N	-3.35505	0.42261	0.205915
C	-2.591562	1.510788	0.005615	C	-2.58991	1.512406	0.215256
N	-1.220321	1.572548	0.001261	N	-1.22223	1.57551	0.250275
C	-0.823285	2.888402	-0.00295	C	-0.82581	2.88783	0.208996
C	-2.008066	3.735259	-0.001558	C	-2.00976	3.737092	0.145503
C	-3.122846	2.873375	0.004011	C	-3.12454	2.8743	0.151805
N	0.42228	3.356888	-0.007449	N	0.421755	3.35657	0.197084
C	1.510119	2.592443	-0.009631	C	1.511587	2.591512	0.207335
N	1.571882	1.221195	-0.008442	N	1.57478	1.223936	0.246868
C	2.872704	3.123719	-0.014283	C	2.873333	3.125916	0.138552
C	3.734586	2.008924	-0.01634	C	3.736032	2.011062	0.133138
C	5.120213	2.187599	-0.021407	C	5.119654	2.190305	0.051808

C	3.355709	4.431291	-0.016436
C	5.615413	3.485423	-0.024021
C	4.742771	4.597155	-0.021201
C	-4.430419	3.356377	0.006393
C	-2.186758	5.120888	-0.005195
C	-3.484585	5.616086	-0.003242
C	-4.596301	4.743439	0.002531
H	-6.683684	-3.67518	0.029723
H	-5.789472	-1.331589	0.023646
H	-2.680758	-5.276931	0.013618
H	-5.277572	2.681649	0.010875
H	-1.332289	5.790386	-0.009576
H	-3.675886	6.684597	-0.006083
H	2.680985	5.278457	-0.014299
H	6.683923	3.676711	-0.027915
H	5.789708	1.333118	-0.023193
H	1.332509	-5.788863	0.007276
H	5.2778	-2.680129	-0.012158
H	3.676105	-6.683076	0.004194
O	5.373361	5.819498	-0.023656
O	5.818872	-5.372504	-0.005011
O	-5.373126	-5.817969	0.025094
O	-5.81865	5.374022	0.003669
Zn	0.000108	0.000761	-0.002336
C	-4.613656	-6.99765	0.017944
C	-4.284932	-7.594549	1.258519
C	-4.299226	-7.589072	-1.228982
C	-3.443085	-8.714311	1.204138
C	-3.4565	-8.708777	-1.189034
C	-2.985239	-9.255989	0.003693
H	-3.136075	-9.195422	2.124544
H	-3.159579	-9.185607	-2.114961
C	6.998531	-4.612993	-0.011437
C	7.596618	-4.286955	1.229279
C	7.588744	-4.295837	-1.258242
C	8.716277	-3.444907	1.175576
C	8.708498	-3.453228	-1.217614
C	9.256816	-2.984493	-0.024388
H	9.198247	-3.139812	2.096164
H	9.184448	-3.154357	-2.14337
C	4.613877	6.999175	-0.01726
C	4.286329	7.59606	-1.258153
C	4.29826	7.590606	1.229361
C	3.444403	8.715802	-1.204571
C	3.455586	8.710321	1.188612
C	2.985435	9.257503	-0.004566
H	3.138231	9.19689	-2.125268
H	3.157816	9.187172	2.114256

C	3.354943	4.43217	0.068568
C	5.613429	3.487509	-0.02024
C	4.740684	4.598791	-0.01129
C	-4.43107	3.355987	0.087925
C	-2.18947	5.12099	0.069855
C	-3.48699	5.614876	0.004272
C	-4.59815	4.741979	0.01386
H	-6.68191	-3.67339	-0.03229
H	-5.79565	-1.33891	0.080009
H	-2.68258	-5.27941	0.094547
H	-5.28038	2.68377	0.092365
H	-1.33972	5.796524	0.060898
H	-3.67417	6.68231	-0.05549
H	2.682888	5.281621	0.072214
H	6.680647	3.674369	-0.0847
H	5.79503	1.340425	0.043253
H	1.339519	-5.79403	0.058702
H	5.280649	-2.6817	0.073587
H	3.673439	-6.6801	-0.06658
O	5.365845	5.819791	-0.09086
O	5.818844	-5.36531	-0.07853
O	-5.36677	-5.81876	-0.04223
O	-5.81958	5.367225	-0.05862
Zn	0.000812	0.00135	0.499729
C	-4.60968	-7.00134	-0.01698
C	-4.36467	-7.61815	1.233822
C	-4.21681	-7.57524	-1.25045
C	-3.52644	-8.74275	1.216371
C	-3.3855	-8.70228	-1.17361
C	-2.99567	-9.2709	0.039324
H	-3.28116	-9.23957	2.14677
H	-3.03285	-9.16803	-2.08544
C	7.002301	-4.60977	-0.04964
C	7.622008	-4.37573	1.201869
C	7.574358	-4.20791	-1.2811
C	8.74842	-3.53984	1.188751
C	8.703382	-3.37972	-1.19993
C	9.275526	-3.00109	0.01489
H	9.247601	-3.30288	2.120056
H	9.167939	-3.02067	-2.10987
C	4.610922	7.003476	-0.05617
C	4.202332	7.577319	-1.28459
C	4.384219	7.621665	1.197452
C	3.375289	8.706731	-1.19729
C	3.54889	8.748551	1.190487
C	3.003917	9.277559	0.020369
H	3.011384	9.172632	-2.10462
H	3.317387	9.246631	2.123767

C	-6.998304	4.614509	0.010973	C	-7.00252	4.611016	-0.02547
C	-7.597083	4.288164	-1.229329	C	-7.57883	4.209002	-1.25486
C	-7.587818	4.297655	1.258184	C	-7.6173	4.37605	1.228271
C	-8.716722	3.446146	-1.174806	C	-8.70651	3.379374	-1.16969
C	-8.707573	3.455006	1.218376	C	-8.74264	3.538671	1.219193
C	-9.256571	2.985998	0.025572	C	-9.27343	2.999481	0.04718
H	-9.199219	3.140856	-2.095054	H	-9.1741	3.020135	-2.078
H	-9.182989	3.156326	2.144469	H	-9.23799	3.300824	2.152323
C	7.14406	-4.885347	2.590359	C	7.183831	-5.05676	2.529109
C	8.146933	-4.541852	3.715864	C	8.216102	-4.81082	3.653954
H	8.17863	-3.468172	3.933192	H	8.275087	-3.75591	3.943864
H	7.833906	-5.048828	4.634969	H	7.90989	-5.37508	4.541335
H	9.16302	-4.878467	3.482793	H	9.219957	-5.14975	3.375528
C	5.767252	-4.351983	3.053093	C	5.828908	-4.52586	3.055241
H	5.748604	-3.256139	3.057274	H	5.837927	-3.43264	3.134988
H	4.944784	-4.701944	2.429196	H	4.986491	-4.81371	2.425704
H	5.570363	-4.693896	4.076623	H	5.644184	-4.93312	4.056769
C	7.101055	-6.43062	2.490556	C	7.104768	-6.59142	2.332152
H	6.388157	-6.770898	1.738166	H	6.369725	-6.87053	1.575713
H	8.088787	-6.829991	2.231863	H	8.079325	-6.99327	2.030075
H	6.809404	-6.856886	3.458329	H	6.823546	-7.07046	3.277851
C	7.12805	-4.904424	-2.612033	C	7.084358	-4.70999	-2.66833
C	8.123582	-4.568587	-3.746327	C	8.059853	-4.29434	-3.79367
H	7.805199	-5.082707	-4.659592	H	7.717239	-4.73388	-4.73668
H	8.153047	-3.496536	-3.971908	H	8.094817	-3.20828	-3.93447
H	9.141405	-4.902628	-3.517157	H	9.078809	-4.65288	-3.61197
C	7.086943	-6.448976	-2.500544	C	7.032543	-6.25837	-2.67447
H	8.076647	-6.845686	-2.245304	H	8.022423	-6.67986	-2.46256
H	6.379184	-6.784227	-1.741065	H	6.329802	-6.64689	-1.93552
H	6.789391	-6.882593	-3.463248	H	6.721843	-6.61404	-3.66434
C	5.747925	-4.375738	-3.070368	C	5.700121	-4.1387	-3.05637
H	4.929344	-4.722659	-2.439679	H	4.893981	-4.52191	-2.43039
H	5.727793	-3.279979	-3.081667	H	5.693345	-3.04449	-2.99078
H	5.545802	-4.724639	-4.090507	H	5.471086	-4.41354	-4.09321
C	10.425443	-2.02601	-0.031605	C	10.45372	-2.05508	0.056187
H	11.057177	-2.155928	0.853721	H	11.08731	-2.24214	0.929526
H	11.053194	-2.164484	-0.918473	H	11.07355	-2.14674	-0.84204
H	10.084261	-0.981867	-0.035895	H	10.12079	-1.01006	0.114509
C	4.883266	7.142126	-2.619389	C	4.695749	7.088606	-2.67537
C	4.350499	5.764224	-3.079527	C	4.120715	5.705465	-3.06166
H	4.701979	4.942948	-2.454916	H	4.506361	4.898295	-2.43852
H	3.254667	5.744619	-3.082287	H	3.026873	5.699944	-2.99003
H	4.691277	5.566206	-4.103216	H	4.389556	5.477096	-4.10023
C	4.537492	8.143138	-3.745833	C	4.274365	8.065954	-3.79698
H	4.873818	9.159774	-3.51476	H	4.63481	9.084357	-3.61596
H	5.043292	7.829103	-4.665244	H	4.708157	7.724245	-4.74298
H	3.463457	8.173842	-3.961566	H	3.187538	8.102006	-3.93143
C	6.428694	7.100499	-2.521353	C	6.244036	7.035294	-2.69106

H	6.827578	8.088863	-2.264325	H	6.667777	8.024476	-2.48039
H	6.770381	6.388787	-1.768468	H	6.636513	6.331121	-1.95557
H	6.854097	6.807988	-3.489248	H	6.593229	6.725668	-3.68355
C	4.908267	7.131255	2.582992	C	5.072025	7.181256	2.520444
C	4.574765	8.12868	3.716332	C	4.833246	8.212505	3.647768
H	3.503088	8.159194	3.943521	H	3.780056	8.271995	3.943758
H	5.090052	7.811285	4.629281	H	5.402346	7.904921	4.531582
H	4.909132	9.145943	3.485148	H	5.171334	9.216385	3.36842
C	6.452648	7.088678	2.469749	C	6.60555	7.10093	2.31531
H	6.786463	6.379704	1.710784	H	6.880049	6.366787	1.556323
H	6.849894	8.077734	2.212834	H	7.006774	8.07557	2.012656
H	6.887092	6.791973	3.432339	H	7.089207	6.817821	3.258091
C	4.378908	5.752249	3.043935	C	4.542438	5.826305	3.047838
H	3.283146	5.733147	3.056657	H	3.449641	5.836332	3.133061
H	4.724258	4.932447	2.41397	H	4.826253	4.984224	2.416019
H	4.728924	5.551234	4.063913	H	4.954504	5.640133	4.047132
C	2.026983	10.426155	0.002676	C	2.058826	10.45622	0.068548
H	2.165721	11.054072	0.889381	H	2.250934	11.08837	0.941835
H	2.156669	11.057717	-0.882811	H	2.145962	11.07739	-0.82921
H	0.982833	10.085	0.007308	H	1.013941	10.12378	0.131987
C	-4.910487	-7.12972	-2.58205	C	-4.73214	-7.08981	-2.6344
C	-4.578242	-8.127281	-3.715642	C	-4.32383	-8.06701	-3.76094
H	-5.094433	-7.809907	-4.628088	H	-4.77237	-7.72783	-4.70094
H	-3.506804	-8.157935	-3.943934	H	-3.23894	-8.09961	-3.91108
H	-4.912481	-9.144488	-3.484024	H	-4.67819	-9.08642	-3.57371
C	-6.454756	-7.08692	-2.467349	C	-6.28062	-7.04202	-2.62682
H	-6.851898	-8.07592	-2.210058	H	-6.69761	-8.03251	-2.40891
H	-6.787769	-6.377894	-1.708082	H	-6.66419	-6.33865	-1.88588
H	-6.890053	-6.790166	-3.429539	H	-6.64607	-6.73442	-3.61409
C	-4.381376	-5.750827	-3.043622	C	-4.16802	-5.70499	-3.03102
H	-4.726021	-4.930905	-2.413424	H	-4.54866	-4.89805	-2.40445
H	-3.285625	-5.731868	-3.057383	H	-3.07334	-5.69478	-2.97429
H	-4.732331	-5.549871	-4.06329	H	-4.45189	-5.47991	-4.06627
C	-4.880585	-7.140601	2.62031	C	-5.0372	-7.17933	2.565162
C	-6.426109	-7.099143	2.523746	C	-6.57341	-7.10489	2.378813
H	-6.768582	-6.387473	1.771176	H	-6.85968	-6.37151	1.623423
H	-6.825132	-8.08755	2.267101	H	-6.97464	-8.0809	2.080649
H	-6.850631	-6.806668	3.492039	H	-7.04673	-6.8238	3.327432
C	-4.347515	-5.762609	3.079815	C	-4.50668	-5.82186	3.08507
H	-3.251682	-5.74291	3.08154	H	-3.41292	-5.8275	3.157391
H	-4.699656	-4.941441	2.455437	H	-4.80137	-4.98154	2.455889
H	-4.687345	-5.564511	4.103803	H	-4.90768	-5.63646	4.088987
C	-4.533593	-8.141496	3.74648	C	-4.78016	-8.20881	3.690066
H	-4.870073	-9.158176	3.515828	H	-5.11839	-9.21412	3.416088
H	-3.459336	-8.172091	3.961116	H	-3.72296	-8.26438	3.972208
H	-5.038482	-7.827433	4.666383	H	-5.33862	-7.90249	4.581068
C	-2.0268	-10.424644	-0.004476	C	-2.04659	-10.4468	0.075478
H	-2.155758	-11.056316	0.881041	H	-2.2262	-11.0799	0.950754

H	-2.166273	-11.052452	-0.891142	H	-2.14287	-11.0678	-0.82144
H	-0.982653	-10.083494	-0.009922	H	-1.00199	-10.1113	0.126371
C	-7.126432	4.906607	2.611575	C	-7.17529	5.05774	2.553874
C	-8.121402	4.571105	3.746463	C	-8.20339	4.810677	3.682281
H	-8.150737	3.499124	3.972399	H	-8.25985	3.755781	3.97276
H	-7.802575	5.085517	4.659408	H	-7.89504	5.375715	4.568425
H	-9.139345	4.905053	3.517691	H	-9.20868	5.147993	3.4071
C	-7.085369	6.451129	2.499641	C	-7.09882	6.592466	2.356395
H	-6.377999	6.786171	1.739708	H	-6.36664	6.872336	1.597453
H	-8.0752	6.847777	2.244799	H	-8.07488	6.993066	2.057516
H	-6.787323	6.885006	3.462075	H	-6.81502	7.072016	3.301069
C	-5.746085	4.37804	3.069389	C	-5.81787	4.528697	3.075433
H	-5.725923	3.282284	3.080907	H	-5.82512	3.435472	3.155278
H	-4.927791	4.724847	2.438264	H	-4.97797	4.81768	2.443032
H	-5.543513	4.727158	4.089364	H	-5.63035	4.936277	4.076306
C	-7.145231	4.886191	-2.590804	C	-7.0949	4.712464	-2.64369
C	-8.148672	4.542373	-3.7157	C	-8.07437	4.296278	-3.76538
H	-7.836102	5.049063	-4.63512	H	-7.73616	4.736895	-4.70948
H	-8.1805	3.468628	-3.93268	H	-8.10849	3.210263	-3.90677
H	-9.164634	4.879082	-3.48222	H	-9.09305	4.653358	-3.57933
C	-5.768654	4.3527	-3.054066	C	-5.71148	4.143167	-3.03759
H	-4.9459	4.702757	-2.430603	H	-4.90334	4.527067	-2.41461
H	-5.750044	3.256854	-3.058041	H	-5.70305	3.048924	-2.97269
H	-5.572215	4.694407	-4.077752	H	-5.48696	4.418931	-4.07517
C	-7.102178	6.43149	-2.491442	C	-7.04505	6.260912	-2.64908
H	-8.089779	6.830929	-2.232352	H	-8.03461	6.681024	-2.433
H	-6.388893	6.771971	-1.739512	H	-6.33986	6.649857	-1.91268
H	-6.811024	6.857495	-3.45948	H	-6.73872	6.617587	-3.63995
C	-10.425184	2.027506	0.033674	C	-10.4501	2.051746	0.092718
H	-11.0576	2.157423	-0.851168	H	-11.0729	2.141918	-0.80361
H	-10.08399	0.983366	0.03769	H	-10.1154	1.007258	0.150629
H	-11.052256	2.165969	0.921022	H	-11.0812	2.238352	0.967939

Data S2. The calculated optimized geometries of dt-ZnPc.

**Optimized geometry of dt-ZnPc (in gas-phase//6-311++G(2d,2p)//Zn/gen/6-31G(d,p)**				**Optimized geometry of relaxed excited state of dt-ZnPc (in DMSO phase// B3LYP/6-31G(d))**			
C	4.58268	-4.73781	-0.00768	C	1.675195	-6.40178	0.125933
C	3.474095	-5.60634	-0.00348	C	0.285226	-6.61665	0.126855
C	4.417714	-3.35663	-0.00903	C	2.208035	-5.10023	0.147681
C	2.18133	-5.11226	-0.00055	C	-0.60488	-5.54148	0.148293
C	3.11476	-2.87279	-0.00601	C	1.305096	-4.04893	0.172347
C	2.002675	-3.73072	-0.00184	C	-0.08146	-4.25211	0.172724
C	2.58669	-1.51115	-0.00613	C	1.518237	-2.58151	0.191939
C	0.819915	-2.88462	0.000488	C	-0.70707	-2.91886	0.191669
N	1.219423	-1.57354	-0.00221	N	0.290166	-1.95848	0.204858
N	-0.42484	-3.34542	0.004549	N	-2.00828	-2.73936	0.173626
N	3.346282	-0.4242	-0.00947	N	2.714925	-2.04307	0.175

C	-1.51178	-2.58582	0.006929	C	-2.59389	-1.5127	0.164959
C	2.885477	0.820557	-0.0094	C	2.923566	-0.6958	0.164749
N	-1.57418	-1.21855	0.005906	N	-1.9753	-0.28999	0.224525
C	-2.87342	-3.11389	0.011232	C	-4.02878	-1.31092	0.063814
C	-3.73135	-2.0018	0.012713	C	-4.23309	0.090585	0.06508
C	-2.88525	-0.81904	0.009246	C	-2.92355	0.695581	0.166254
C	-3.35725	-4.41685	0.013599	C	-5.093	-2.20841	-0.03122
C	-5.11288	-2.18045	0.016765	C	-5.52769	0.613137	-0.02955
C	-4.73843	-4.58181	0.01754	C	-6.3807	-1.66947	-0.12345
C	-5.60696	-3.47322	0.019174	C	-6.5951	-0.27182	-0.12169
N	-3.34606	0.42572	0.009307	N	-2.7149	2.042844	0.177233
C	-2.58647	1.512668	0.005975	C	-1.5182	2.581259	0.193883
N	-1.2192	1.575061	0.002048	N	-0.29012	1.958205	0.205516
C	-0.81969	2.886139	-0.00065	C	0.707106	2.918617	0.192787
C	-2.00245	3.73224	0.001676	C	0.081478	4.25188	0.175309
C	-3.11454	2.874305	0.005855	C	-1.30507	4.048693	0.175551
N	0.425061	3.346941	-0.00471	N	2.008306	2.739133	0.174188
C	1.512007	2.587343	-0.00709	C	2.593906	1.512481	0.164621
N	1.574403	1.22007	-0.00606	N	1.975343	0.289742	0.224022
C	2.873638	3.11541	-0.0114	C	4.028738	1.31074	0.062617
C	3.731573	2.003319	-0.01288	C	4.23305	-0.09076	0.063147
C	5.113104	2.181973	-0.01694	C	5.527597	-0.61327	-0.03246
C	3.357474	4.418367	-0.01377	C	5.092913	2.208279	-0.03261
C	5.607181	3.474742	-0.01936	C	6.594952	0.271726	-0.12482
C	4.738652	4.583333	-0.01772	C	6.380559	1.669369	-0.12581
C	-4.41749	3.358146	0.008874	C	-2.20803	5.100028	0.152621
C	-2.18111	5.113776	0.000388	C	0.604894	5.541273	0.151866
C	-3.47388	5.607858	0.003312	C	-0.28523	6.616465	0.132143
C	-4.58246	4.739327	0.007515	C	-1.6752	6.401599	0.132007
H	-6.67113	-3.6611	0.022291	H	-7.61542	0.090485	-0.1958
H	-5.78086	-1.33121	0.017953	H	-5.69891	1.685244	-0.03106
H	-2.68539	-5.26046	0.012336	H	-4.92838	-3.27906	-0.03394
H	-5.26109	2.686287	0.01208	H	-3.27724	4.926725	0.150312
H	-1.33187	5.78176	-0.00283	H	1.676751	5.712668	0.149266
H	-3.66175	6.672035	0.002415	H	0.078071	7.638918	0.115268
H	2.685616	5.261977	-0.01251	H	4.928293	3.278927	-0.03476
H	6.671355	3.662614	-0.02249	H	7.615227	-0.09055	-0.19967
H	5.781088	1.332728	-0.01814	H	5.698811	-1.68538	-0.03457
H	1.332089	-5.78024	0.002663	H	-1.67674	-5.71288	0.146178
H	5.26132	-2.68477	-0.01224	H	3.277246	-4.92693	0.144814
H	3.661971	-6.67052	-0.00258	H	-0.07808	-7.63909	0.109096
O	5.367987	5.803555	-0.02033	O	7.523586	2.4248	-0.22504
O	5.8029	-5.36715	-0.01011	O	2.441071	-7.53868	0.101615
O	-5.36776	-5.80204	0.020132	O	-7.52378	-2.42486	-0.22236
O	-5.80268	5.368667	0.009953	O	-2.44108	7.538538	0.10962
Zn	0.000112	0.00076	-2.4E-05	Zn	0.00007	-0.0002	0.528602
C	-4.6088	-6.97996	0.018098	C	-7.44367	-3.82692	-0.24057
C	-4.28233	-7.57233	1.255759	C	-7.55409	-4.52342	0.987337

C	-4.28991	-7.57304	-1.22119	C	-7.35991	-4.48094	-1.49375
C	-3.4382	-8.68465	1.207072	C	-7.37446	-5.91334	0.930274
C	-3.44548	-8.68535	-1.17698	C	-7.18746	-5.87246	-1.45643
C	-2.97566	-9.22506	0.013773	C	-7.14847	-6.59676	-0.26476
H	-3.13571	-9.16108	2.125523	H	-7.42132	-6.49497	1.842542
H	-3.14859	-9.16231	-2.09699	H	-7.08999	-6.42276	-2.38398
C	6.980815	-4.60819	-0.01487	C	3.843918	-7.44894	0.094066
C	7.57721	-4.28349	1.221323	C	4.526374	-7.48192	1.334099
C	7.569859	-4.28752	-1.25562	C	4.510524	-7.4349	-1.15495
C	8.68938	-3.43929	1.17022	C	5.915803	-7.29753	1.28128
C	8.682306	-3.44315	-1.21383	C	5.900556	-7.25217	-1.11272
C	9.225899	-2.97504	-0.02417	C	6.61125	-7.13898	0.082371
H	9.168799	-3.13813	2.087548	H	6.487497	-7.28647	2.200874
H	9.156269	-3.14495	-2.13496	H	6.460586	-7.20616	-2.03838
C	4.609035	6.981488	-0.01828	C	7.44348	3.826873	-0.24248
C	4.282566	7.573862	-1.25594	C	7.358986	4.481542	-1.49527
C	4.290143	7.574564	1.221012	C	7.554622	4.522745	0.985716
C	3.438424	8.686184	-1.20724	C	7.186564	5.873042	-1.45714
C	3.445715	8.686866	1.176818	C	7.374971	5.912689	0.929472
C	2.975891	9.226584	-0.01394	C	7.148287	6.596728	-0.26508
H	3.135923	9.162611	-2.12568	H	7.08855	6.423817	-2.38435
H	3.148833	9.163823	2.096823	H	7.422367	6.493853	1.84201
C	-6.98061	4.609721	0.014719	C	-3.84394	7.448851	0.103874
C	-7.57705	4.285073	-1.22146	C	-4.5122	7.436228	-1.14427
C	-7.56962	4.289017	1.255485	C	-4.52476	7.480462	1.344847
C	-8.68922	3.440869	-1.17035	C	-5.90217	7.253483	-1.10042
C	-8.68206	3.444657	1.213707	C	-5.91426	7.296147	1.293643
C	-9.2257	2.976588	0.024042	C	-6.61129	7.138962	0.095478
H	-9.16867	3.139723	-2.08766	H	-6.46342	7.208514	-2.0254
H	-9.156	3.14642	2.134836	H	-6.48475	7.284072	2.213974
C	7.129215	-4.88311	2.581624	C	3.841949	-7.7992	2.693466
C	8.126856	-4.53068	3.70507	C	4.887601	-8.01333	3.812265
H	8.154891	-3.46103	3.914504	H	5.448454	-7.10005	4.039947
H	7.812252	-5.03118	4.620771	H	4.366521	-8.31169	4.728325
H	9.139025	-4.86531	3.477569	H	5.602278	-8.80558	3.564323
C	5.750998	-4.36029	3.04028	C	2.912993	-6.65936	3.174465
H	5.729249	-3.27008	3.054738	H	3.442316	-5.69963	3.190905
H	4.938965	-4.70505	2.409709	H	2.023553	-6.547	2.553808
H	5.550359	-4.71143	4.0541	H	2.574683	-6.87272	4.195703
C	7.099484	-6.42657	2.485603	C	3.038068	-9.11876	2.580573
H	6.383587	-6.77619	1.748065	H	2.242602	-9.05221	1.836602
H	8.083218	-6.81458	2.217431	H	3.697191	-9.95099	2.306145
H	6.825952	-6.84932	3.453944	H	2.582445	-9.35754	3.549195
C	7.113804	-4.89143	-2.61134	C	3.809059	-7.70057	-2.51676
C	8.10512	-4.54307	-3.74164	C	4.84069	-7.87339	-3.65557
H	7.785206	-5.04661	-4.65383	H	4.308033	-8.13725	-4.57556
H	8.132224	-3.47415	-3.95489	H	5.399372	-6.95244	-3.85603
H	9.118479	-4.87718	-3.51872	H	5.557679	-8.67461	-3.4461

C	7.084	-6.43455	-2.51023	C	3.005094	-9.02286	-2.44347
H	8.069074	-6.82211	-2.24635	H	3.66669	-9.86533	-2.20914
H	6.372118	-6.78157	-1.7676	H	2.21934	-8.98395	-1.68732
H	6.804895	-6.86025	-3.47569	H	2.53685	-9.2246	-3.41449
C	5.733189	-4.36945	-3.0637	C	2.87586	-6.54267	-2.94324
H	4.924708	-4.71151	-2.42712	H	1.994359	-6.45285	-2.30783
H	5.711973	-3.27928	-3.08184	H	3.406324	-5.58352	-2.93014
H	5.52637	-4.72402	-4.07508	H	2.524763	-6.71724	-3.96752
C	10.38637	-2.01093	-0.02915	C	8.100228	-6.87945	0.077788
H	11.01581	-2.14009	0.851054	H	8.587267	-7.32226	0.953
H	11.01015	-2.14238	-0.91304	H	8.575591	-7.28674	-0.82078
H	10.0359	-0.97585	-0.02937	H	8.314378	-5.80242	0.097962
C	4.880265	7.121446	-2.61562	C	7.541595	3.764923	-2.86291
C	4.356923	5.741665	-3.06896	C	6.358337	2.831185	-3.21145
H	4.702566	4.931774	-2.43612	H	6.301133	1.95899	-2.55983
H	3.266692	5.71981	-3.08189	H	5.403345	3.365925	-3.15108
H	4.706722	5.537648	-4.08256	H	6.473785	2.466707	-4.23948
C	4.526118	8.115337	-3.74185	C	7.650669	4.783674	-4.02099
H	4.860966	9.128285	-3.51816	H	8.465481	5.499468	-3.86715
H	5.025338	7.797772	-4.65722	H	7.857667	4.24019	-4.94919
H	3.456168	8.142571	-3.94984	H	6.721478	5.34414	-4.17256
C	6.423856	7.092167	-2.52173	C	8.863415	2.956848	-2.85803
H	6.812157	8.076787	-2.25724	H	9.720355	3.617807	-2.68129
H	6.77461	6.378654	-1.78242	H	8.865939	2.180342	-2.09138
H	6.845245	6.815594	-3.4898	H	9.006401	2.47653	-3.83361
C	4.895956	7.122889	2.57736	C	7.950693	3.850637	2.330475
C	4.549226	8.117861	3.70495	C	8.236931	4.906275	3.423403
H	3.480619	8.145671	3.919642	H	7.341665	5.474261	3.699798
H	5.054065	7.80088	4.617443	H	8.587531	4.392953	4.325258
H	4.883037	9.130491	3.47829	H	9.016101	5.614289	3.120795
C	6.438934	7.092711	2.474185	C	9.257503	3.039214	2.145696
H	6.784901	6.378504	1.7333	H	9.141831	2.237077	1.415013
H	6.826154	8.076943	2.206686	H	10.07445	3.691438	1.814581
H	6.865972	6.816612	3.439912	H	9.552863	2.591772	3.102496
C	4.374569	5.743767	3.034931	C	6.838797	2.931698	2.889698
H	3.284423	5.722615	3.054561	H	5.884811	3.466707	2.962376
H	4.715797	4.933195	2.400572	H	6.682478	2.039096	2.283451
H	4.730458	5.540268	4.046523	H	7.114509	2.600116	3.89821
C	2.011789	10.38707	-0.01132	C	6.896327	8.087052	-0.27
H	2.144728	11.01389	0.870186	H	7.394763	8.581	0.570784
H	2.139447	11.01347	-0.89391	H	7.249739	8.550545	-1.19715
H	0.976704	10.03661	-0.00814	H	5.823695	8.307695	-0.18577
C	-4.89572	-7.12139	-2.57754	C	-7.54333	-3.76362	-2.8609
C	-4.549	-8.1164	-3.7051	C	-7.65305	-4.78177	-4.01945
H	-5.05383	-7.79943	-4.6176	H	-7.86065	-4.23781	-4.94724
H	-3.4804	-8.14423	-3.91979	H	-6.72391	-5.34209	-4.17188
H	-4.88283	-9.12901	-3.47842	H	-8.46771	-5.4977	-3.86548
C	-6.4387	-7.09119	-2.47435	C	-8.86518	-2.95559	-2.85484

H	-6.82593	-8.07542	-2.20683	H	-9.722	-3.61667	-2.67798
H	-6.78465	-6.37697	-1.73348	H	-8.86729	-2.17949	-2.08777
H	-6.86574	-6.81511	-3.44008	H	-9.00873	-2.47475	-3.83008
C	-4.37432	-5.74228	-3.03515	C	-6.3603	-2.82967	-3.20963
H	-4.71555	-4.93169	-2.40081	H	-6.30275	-1.95781	-2.5576
H	-3.28418	-5.72114	-3.05478	H	-5.40526	-3.36441	-3.15008
H	-4.73021	-5.53881	-4.04674	H	-6.47634	-2.46466	-4.23741
C	-4.88005	-7.11991	2.615434	C	-7.94939	-3.852	2.332667
C	-6.42364	-7.09058	2.521501	C	-9.25626	-3.04041	2.14901
H	-6.77434	-6.37704	1.782193	H	-9.14093	-2.2379	1.418681
H	-6.81197	-8.07518	2.25699	H	-10.0734	-3.69242	1.817992
H	-6.84504	-6.81401	3.489566	H	-9.55108	-2.59345	3.106199
C	-4.35667	-5.74016	3.068814	C	-6.83715	-2.93342	2.891778
H	-3.26644	-5.71834	3.081767	H	-5.88315	-3.4685	2.963647
H	-4.70228	-4.93024	2.435988	H	-6.68113	-2.04049	2.285931
H	-4.70648	-5.53616	4.082419	H	-7.1123	-2.60237	3.90062
C	-4.52596	-8.11384	3.741643	C	-8.2351	-4.9082	3.425192
H	-4.86085	-9.12677	3.517933	H	-9.01449	-5.616	3.122644
H	-3.45601	-8.14113	3.949644	H	-7.33972	-5.4764	3.700784
H	-5.02517	-7.79627	4.65702	H	-8.58516	-4.39533	4.327515
C	-2.01155	-10.3855	0.011196	C	-6.89651	-8.08708	-0.2706
H	-2.13981	-11.0124	0.893362	H	-7.3945	-8.58147	0.570185
H	-2.14389	-11.0119	-0.87074	H	-7.25039	-8.55009	-1.19781
H	-0.97647	-10.0351	0.008922	H	-5.82383	-8.30776	-0.18704
C	-7.11349	4.892867	2.611212	C	-3.83857	7.796162	2.703701
C	-8.10475	4.544518	3.741565	C	-4.8828	8.009117	3.824054
H	-8.13188	3.4756	3.954794	H	-5.44338	7.09561	4.051483
H	-7.78476	5.048028	4.653743	H	-4.36055	8.306495	4.739768
H	-9.11811	4.878672	3.518717	H	-5.59778	8.801647	3.577854
C	-7.08362	6.435991	2.510152	C	-3.03472	9.115789	2.591331
H	-6.37177	6.783008	1.767495	H	-2.24017	9.050043	1.84631
H	-8.0687	6.823598	2.246334	H	-3.69412	9.948386	2.31867
H	-6.80445	6.861644	3.475606	H	-2.5779	9.353418	3.559669
C	-5.73286	4.370821	3.063482	C	-2.90914	6.655673	3.18223
H	-5.7117	3.280648	3.081596	H	-3.43857	5.695999	3.198277
H	-4.9244	4.712857	2.426864	H	-2.02052	6.543896	2.560295
H	-5.52597	4.72536	4.074859	H	-2.56949	6.86785	4.203268
C	-7.12908	4.884724	-2.58176	C	-3.8125	7.703428	-2.50669
C	-8.12665	4.532183	-3.70525	C	-4.84559	7.877267	-3.64401
H	-7.81208	5.032739	-4.62093	H	-4.31414	8.142132	-4.56441
H	-8.15453	3.462536	-3.91471	H	-5.40441	6.956451	-3.8447
H	-9.13887	4.866681	-3.47778	H	-5.56242	8.678174	-3.43283
C	-5.7508	4.362031	-3.04037	C	-2.87962	6.546144	-2.93554
H	-4.93882	4.706864	-2.40977	H	-1.99732	6.455815	-2.30131
H	-5.72895	3.27182	-3.05482	H	-3.4099	5.58689	-2.92281
H	-5.55016	4.713179	-4.05418	H	-2.52982	6.721855	-3.96006
C	-7.09951	6.42818	-2.48578	C	-3.00866	9.025776	-2.43301
H	-8.08329	6.816098	-2.21763	H	-3.67009	9.86788	-2.19691

H	-6.38366	6.777901	-1.74823	H	-2.22192	8.986169	-1.67792
H	-6.82601	6.850941	-3.45412	H	-2.54171	9.228646	-3.40442
C	-10.3862	2.012493	0.02906	C	-8.10028	6.879467	0.092576
H	-11.0152	2.141063	-0.85157	H	-8.57686	7.287965	-0.8048
H	-10.0357	0.977403	0.030167	H	-8.31443	5.802416	0.111605
H	-11.0104	2.144533	0.912532	H	-8.58612	7.32113	0.969038

3. TDDFT calculation

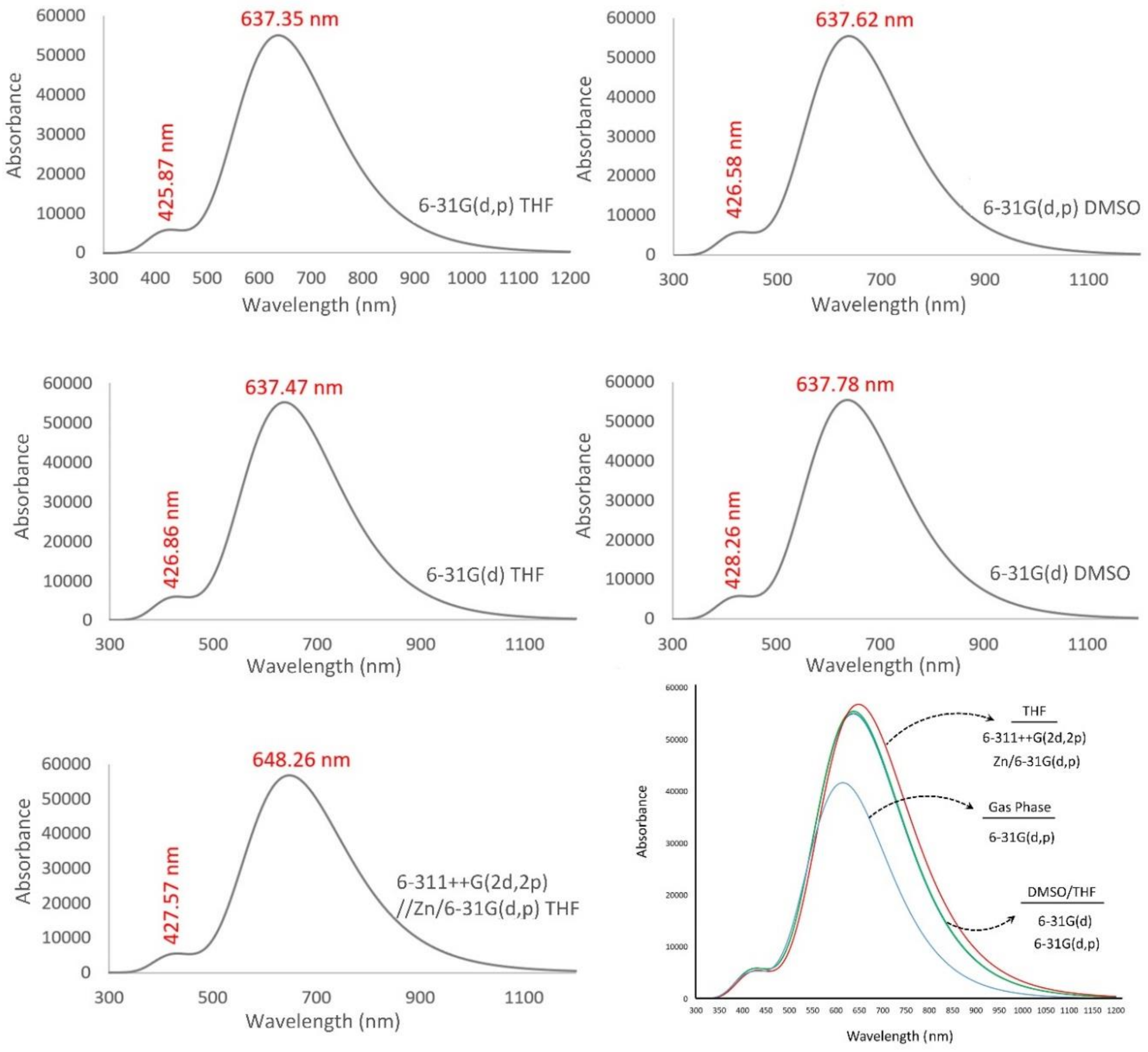

Figure S1. The calculated UV-vis spectra of dt-ZnPc.

Table S1. Vertical Excitation TDDFT calculation on Pcs (eV//nm) by B3lyp/6-31G(d)

Comp.	Solvent	S1 (eV//nm)	S2 (eV//nm)	T1 (eV//nm)	T2 (eV//nm)	S1-T1 (eV//nm)	S1-T2 (eV//nm)
**Pc**	-	2.065//600.39	2.089// 593.52	1.023//1212.39	1.190//1041.60	1.043//1188.73	0.875//1416.96
	THF	1.982//625.49	1.984//625.03	1.003//1235.81	1.182//1048.56	0.979//1266.44	0.800//1549.80
	DMSO	1.977//627.17	1.982//625.69	1.000//1240.29	1.181//1049.74	0.977//1269.03	0.796//1557.59
**dt-H_2Pc**	-	1.905//651.02	1.931//642.00	1.006//1232.93	1.168//1061.21	0.899//1379.13	0.736//1684.57
	THF	1.903//651.52	1.929//642.60	0.985//1258.41	1.154//1074.14	0.918//1350.59	0.749//1655.33
	DMSO	1.898//653.18	1.925//644.03	0.982//1263.22	1.152//1076.39	0.917//1352.06	0.746//1661.98
**dt-ZnPc**	THF	1.945//637.47	2.905//426.86	1.115//1111.80	2.591//478.57	0.830//1493.79	-0.646//1919
	DMSO	1.944//637.78	2.895//428.26	1.116//1110.93	2.588//479.11	0.828//1497.39	-0.644//1925

Table S2. Vertical Excitation TDDFT calculation on Pcs (eV//nm) by B3lyp/6-311++G(2d,2p)//Zn/6-31G(d,p)

Comp.	Solvent	S1 (eV//nm)	S2 (eV//nm)	T1 (eV//nm)	T2 (eV//nm)	S1-T1 (eV//nm)	S1-T2 (eV//nm)
**dt-ZnPc**	THF	1.913//648.26	2.900//427.57	1.095//1132.57	2.584//479.85	0.818//1515.70	-0.671//1847

Table S3. Adiabatic Excitation TDDFT calculation on Pcs (eV//nm) by B3lyp/6-31G(d)

Comp.	Solvent	S1 (eV//nm)	S2 (eV//nm)	T1 (eV//nm)	T2 (eV//nm)	S1-T1 (eV//nm)	S1-T2 (eV//nm)
**dt-ZnPc**	THF	1.699//729.87	2.699//459.36	0.839//1478.54	1.318//940.44	0.860//1441.68	0.380//3262.74
	DMSO	1.663//745.43	2.693//460.39	0.839//1477.91	1.319//939.89	0.824//1504.66	0.344//3604.19

Table S4. Spin-orbit coupling (SOC)-TDDFT calculation on Pcs (eV//nm) by B3lyp/6-31G(d)

Comp.	Solvent	S1 (eV//nm)	S2 (eV//nm)	T1 (eV//nm)	T2 (eV//nm)	S1-T1 (eV//nm)	S1-T2 (eV//nm)
**dt-H_2Pc**	THF	1.895//654.27	2.669//464.53	0.976//1270.33	1.146//1.146	0.919//1349.12	0.749//1655
**dt-ZnPc**	THF	1.931//642.07	2.886//429.61	1.101//1126.10	2.565//483.37	0.830//1493.78	-0.634//1956

References

1. Güzel E, Çetin Ş, Günsel A, et al (2018) Comparative studies of photophysical and electrochemical properties of sulfur-containing substituted metal-free and metallophthalocyanines. Research on Chemical Intermediates 44:971–989.
2. Güzel E, Arslan BS, Atmaca GY, et al (2019) High Photosensitized Singlet Oxygen Generating Zinc and Chloroindium Phthalocyanines Bearing (4-isopropylbenzyl)oxy Groups as Potential Agents for Photophysicochemical Applications. ChemistrySelect 4:515–520.
3. Maree MD, Nyokong T, Suhling K, Phillips D (2002) Effects of axial ligands on the photophysical properties of silicon octaphenoxyphthalocyanine. J Porphyr Phthalocyanines 06:373–376.
4. Ogunsipe A, Chen J, Nyokong T (2004) Photophysical and photochemical studies of zinc (II) phthalocyanine derivatives—effects of substituents and solvents. New Journal of Chemistry 28:822–827.
5. Brannon JH, Magde D (1980) Picosecond laser photophysics. Group 3A phthalocyanines. J Am Chem Soc 102:62–65. https://doi.org/10.1021/ja00521a011
6. Seotsanyana-Mokhosi I, Kuznetsova N, Nyokong T (2001) Photochemical studies of tetra-2,3-pyridinoporphyrazines. J Photochem Photobiol A Chem 140:215–222.
7. Ogunsipe A, Nyokong T (2005) Photophysical and photochemical studies of sulphonated non-transition metal phthalocyanines in aqueous and non-aqueous media. 173:211–220.

8. Brannon JH, Magde D (1980) Picosecond laser photophysics. Group 3A phthalocyanines. J Am Chem Soc 102:62–65.
9. Seotsanyana-Mokhosi I, Kuznetsova N, Nyokong T (2001) Photochemical studies of tetra-2,3-pyridinoporphyrazines. J Photochem Photobiol A Chem 140:215–222.
